# Auto-Adhesion Potential of Extraocular Aqp0 during Teleost Development

**DOI:** 10.1371/journal.pone.0154592

**Published:** 2016-05-06

**Authors:** François Chauvigné, Per Gunnar Fjelldal, Joan Cerdà, Roderick Nigel Finn

**Affiliations:** 1 Department of Biology, Bergen High Technology Centre, University of Bergen, 5020 Bergen, Norway; 2 Institute of Marine Research, Nordnes, 5817 Bergen, Norway; 3 Institut de Recerca i Tecnologia Agroalimentàries (IRTA)-Institut de Ciències del Mar, Consejo Superior de Investigaciones Científicas (CSIC), 08003 Barcelona, Spain; University of Bari Aldo Moro, ITALY

## Abstract

AQP0 water channels are the most abundant proteins expressed in the mammalian lens fiber membranes where they are essential for lens development and transparency. Unlike other aquaporin paralogs, mammalian AQP0 has a low intrinsic water permeability, but can form cell-to-cell junctions between the lens fibers. It is not known whether the adhesive properties of AQP0 is a derived feature found only in mammals, or exists as a conserved ancestral trait in non-mammalian vertebrates. Here we show that a tetraploid teleost, the Atlantic salmon, expresses four Aqp0 paralogs in the developing lens, but also expresses significant levels of *aqp0* mRNAs and proteins in the epithelia of the pronephros, presumptive enterocytes, gill filament and epidermis. Quantitative PCR reveals that *aqp0* mRNA titres increase by three orders of magnitude between the onset of somitogenesis and pigmentation of the eye. Using *in situ* hybridization and specific antisera, we show that at least two of the channels (Aqp0a1, -0b1 and/or -0b2) are localized in the extraocular basolateral and apical membranes, while Aqp0a2 is lens-specific. Heterologous expression of the Aqp0 paralogs in adhesion-deficient mouse fibolast L-cells reveals that, as for human AQP0, each intact salmon channel retains cell-to-cell adhesive properties. The strongest Aqp0 interactions are auto-adhesion, suggesting that homo-octamers likely form the intercellular junctions of the developing lens and epithelial tissues. The present data are thus the first to show the adhesion potential of Aqp0 channels in a non-mammalian vertebrate, and further uncover a novel extraocular role of the channels during vertebrate development.

## Introduction

AQP0 is a member of a large family of water channel proteins (aquaporins), which in vertebrates consists of 17 subfamilies (Aqp0 to -16; [1,2]). When assembled as tetramers and inserted in biological membranes, aquaporins passively transport water or other small, uncharged molecules down their concentration gradients [3,4]. Biophysical and crystallographic characterisation of mammalian AQP0 has revealed that it is a functional water channel, albeit with a low intrinsic transport of water through the pore [5–8]. Both Ca^2+^ ions, which act through calmodulin, and pH have been shown to modulate the water permeability by promoting open and closed pore states, with high [Ca^2+^] and [H^+^] inducing pore closure [9–13]. Mammalian AQP0 also functions as a cell-to-cell adhesion molecule [14–18], which, apart from a short AQP4-M23 isoform [19,20], and a distantly related big brain ortholog found in arthropods [21–23], is unusual for aquaporins in general. Based upon *in vitro* models, a switch between a water permeation state and a purely intercellular adhesion function is thought to occur when the intracellular carboxy terminal region is cleaved to promote double-layered arrays of AQP0 octamers in junctional microdomains of the inner lens fibers [24–28]. However, it has also been shown that intact AQP0 can perform cell-to-cell adhesion [17]. An alternative suggestion for the low permeation property of mammalian AQP0 is that junction formation rather than cleavage of the carboxy teminus leads to pore closure and thus loss of water transport activity [16,29]. It has yet to be established whether Aqp0 water channels that are efficient water transporters are also capable of cell-to-cell adhesion.

Although it is now known that AQP0 is found in all vertebrates [1,30], it was originally identified as the major intrinsic protein (MIP) of the lens fiber due to its high accummulation in bovine lens membranes [31–34]. Studies of AQP0 in other mammals confirmed the high titres of the channel in the lens fiber membranes, and revealed that its presence is essential for lens development and transparency [24,35–40]. The expression of AQP0 was thus initially thought to be exclusive to the lens fiber membranes [41] where it has been shown to participate in regulating the ocular lens refractive index gradient and the biomechanics of focal accommodation [42,43]. To date, ontogenetic studies of mammalian embryos have so far only detected AQP0 expression in the lens and not in other tissues [44,45]. In adults, however, low level expression has now been detected in the bipolar and ganglion cells of the retina [46–48], and the somatic steroidogenic and germ cells of the testis [49–51].

Until recently, much less was known concerning the biophysical properties and expression sites of non-mammalian orthologs of AQP0. Studies of Aqp0 channels in zebrafish (*Danio rerio*) revealed, however, that there are two paralogs (Aqp0a, -0b), which were reported to differ in their water transport activities [52,53]. This discrepancy in channel activity has recently been resolved by the identification of an alternative Aqp0b allele (Ser^19^ instead of Gly^19^) which abolishes water transport activity [30]. It has thus been established that both Aqp0a and -0b are functional and efficient water transporters. As in mammals, an inherent sensitivity to Ca^2+^ and pH has also been observed in teleost Aqp0 channels [10,30,54]. The most recent investigation established that teleost Aqp0a-type channels display highest water transport activities at alkaline pH, while mammalian AQP0 and some teleost Aqp0b-type channels transport water most efficiently at acidic pH [30]. A more complex regulation is found in tetraploid Atlantic salmon (*Salmo salar*), which expresses equally high mRNA titres of tetraparalogous *aqp0* channels (*aqp0a1*, *-0a2*, *-0b1*, *-0b2*) in the lens, with each channel displaying a unique pH sensitivity when heterologously expressed in *Xenopus laevis* oocytes [30]. To date, however, it remains to be established whether any of the teleost Aqp0 channels possesses the cell-to-cell adhesion property of the mammalian ortholog.

Localization studies of Aqp0 in non-mammalian vertebrates initially supported the selective expression of duplicated Aqp0a and -0b channels in the lens of teleosts, including embryos and larvae [52,54,55]. However, other reports indicated that significant levels of *aqp0b* mRNA are found in the ovary of adult teleosts [53,56,57], while Aqp0a proteins are also localized in the Sertoli cells of the testis [58]. More recently, these latter findings have been supported by observations of the extraocular expression of the four Atlantic salmon *aqp0* mRNAs in tissues such as the brain, gills, kidney, mid intestine, rectum, ovary, and testis [30]. To test for the earliest signs of extraocular expression, we examined the pattern of *aqp0a1*, -*0a2*, *-0b1* and *-0b2* mRNA expression during embryonic and larval development and raised antibodies in rabbits against the carboxy terminus of the “a”- and “b”-type channels to identify the localization of the salmon Aqp0 proteins. To decipher whether a dosage effect occurs following triploidisation, which leads to sterility [59], we compared the ontogenetic expression between diploid (2n) and triploid (3n) siblings. These experiments confirmed the expression of the paralogs in the lens fibers, but further revealed for the first time in any vertebrate that Aqp0 channels are highly accumulated in the basolateral or apical membranes of kidney, intestine, gill and skin epithelia. To establish a possible role for the extraocular expression of the Aqp0 channels, we tested the cell-to-cell adhesion potential of each paralog in adhesion-deficient mouse fibroblast L-cells.

## Materials and Methods

### Animals

Two series of 2n and 3n Atlantic salmon embryos and larvae of the AquaGen strain (http://aquagen.no/en/) were incubated at the Institute of Marine Research in Matre near Bergen, Norway, in darkness under ambient temperature conditions, with a mean ± 1 standard deviation of 4.98 ± 0.42°C between November and May. Triploidy was induced by inhibiting the dissociation of the second polar body during the second meiotic division by applying 9.5 Kpsi of pressure for 5 min at 30 min after fertilisation as described previously [60].

Samples were collected regularly over the 6 month period where embryos were either frozen in liquid nitrogen and stored at -80°C, or fixed in 4% phosphate-buffered parafomaldehyde (137 mM NaCl, 2.7 mM KCl, 100 mM Na_2_HPO_4_, 2 mM KH_2_PO_4_, pH 7.4) or Bouin´s fluid (75 ml saturated aqueous picric acid; 25 ml 37% formaldehyde; 5 ml glacial acetic acid) for subsequent analyses. To obtain better penetration of fixative, embryos were dissected out of their chorions from 250 day degrees onwards. All sampling procedures were conducted in accordance with the regulations by the governmental Norwegian Animal Research Authority, NARA (http://www.fdu.no/fdu/) and were specifically approved by the ethics committee of the Institute of Marine Research.

### Primary antibodies and reagents

Antisera were raised in rabbits against synthetic peptides corresponding to the carboxy terminal amino acid residues of Aqp0a1 and -0a2 (SERMAILKGTRPPEAESQQD) and Aqp0b1 and -b2 (SERLATLKGSRPPETETQQD) at Agrisera (Vännäs, Sweden). Half of the antiserum was affinity-purified against the synthetic peptide, and the specificity was confirmed by ELISA and Western blots of total membrane extracts from *X*. *laevis* oocytes injected with *aqp0a1*, *-0a2*, *-0b1* or *-0b2* cRNA (see [30] for details). The mouse monoclonal antibody against Na^+^-K^+^ ATPase was purchased from the Developmental Studies Hybridoma Bank (ATP1A1 Antibody (a5); DSHB, University of Iowa). The mouse monoclonal antibody against α-tubulin was purchased from Sigma-Aldrich (Clone DMA1, ref. T9026). All other reagents and kits were purchased from Life technologies unless stated otherwise.

### Histological analysis

Embryos collected at different stages of development were fixed in Bouin’s fluid for 16 h at room temperature and were embedded in Technovit 7100 (glycol methacrylate embedding kit, Kulzer) following the manufacter´s instructions. Sections of ~3 μm in thickness were attached to UltraStick/UltraFrost Adhesion slides (Electron Microscopy Sciences, USA) and stained with Toluidine Blue. Alternatively, fixed embryos were mounted in paraffin and sections of 8 μm were counterstained with hematoxylin and eosin as described previously [58].

### Immunofluorescence microscopy

Embryos that were fixed in PFA for 6 h were washed, dehydrated, and embedded in paraffin as previously described [58]. Sections of ~8 μm in thickness were attached to UltraStick/UltraFrost Adhesion slides (Electron Microscopy Sciences, USA) and rehydrated before permeabilization with 0.5% Triton X-100 for 5 min at room temperature. Sections were blocked in 5% goat serum and 0.1% bovine serum albumin (BSA) in phosphate buffered saline (PBS) with 0.1% Tween-20 (PBST) for 1 h before incubation with the antibodies overnight at 4°C in PBST at 1:600 for non-purified and affinity-purified anti-Aqp0 antibodies and 1:1000 for Na^+^-K^+^ ATPase antisera. Slides mounted with adjacent sections were incubated with the antibodies preadsorbed with the immunizing peptides as negative controls. After washing, sections were exposed to a Goat-Alexa 488-coupled anti-rabbit IgG secondary antibody (1:1000; Life Technologies Corp., A-11008) and Goat-Alexa 555-coupled anti-mouse IgG secondary antibody (1:1000; Life Technologies Corp., A-21422) for 1 h at room temperature. The membranes and extracellular matrix were counterstained with wheat germ agglutinin, Alexa Fluor® 647 Conjugate (WGA, 1:3000, Life Technologies Corp., W32466) diluted in PBS for 10 min., washed and the nuclei were counterstained with 4’,6-diamidino-2-phenylindole (DAPI, Sigma-Aldrich, D9564) at 1:3000 in PBS for 3 min, and slides were mounted with fluoromount aqueous anti-fading medium. Sections were examined and photographed with a Zeiss Axio Imager Z1/ApoTome fluorescence microscope (Carl Zeiss Corp.), with the Aqp0-Alexa 488 signal acquired in green, the Na^+^-K^+^ ATPase-Alexa 555 signal acquired in red, the DAPI acquired in blue and the WGA acquired in white. Images from control sections were taken with the same fluorescence intensity and exposure as those used for the positives.

### *In situ* hybridization (ISH)

ISH was carried out on stage 9 and 11 embryos fixed for 16 h at 4°C in PFA. The digoxigenin (DIG)-labeled riboprobes specific for *aqp0a1*, *-0a2* and *-0b1/2* were designed in the 3’UTR or 5’UTR (see S1 Fig). ISH was performed as described previously for trout embryos and larvae [61].

### Protein extraction

Ectopic expression of salmon Aqp0a1, -0a2, -0b1, and -0b2 in *Xenopus laevis* oocytes was carried out as previously described [23,30]. Each embryo was dissociated with a glass dounce homogenizer in ice-cold lens lysis buffer (LLB) containing 50 mM NaCl, 5 mM Tris-HCl, pH 8, 4 M Urea, 20 mM NaOH, 0.5% Triton X-100, 1% NP-40, 5 mM EDTA, 5 mM EGTA, EDTA-free protease inhibitors (Roche Diagnostics), incubated for 15 min on ice and centrifuged at 14000 x *g* for 10 min at 4°C. One aliquot of supernatant was removed to determine the protein concentration with a Nanodrop (ND1000-V3) using the LLB as blank, and the rest of the supernatant was mixed with 2x Laemmli sample buffer and 100 μM dithiothreitol (DTT), heated at 95°C for 10 minutes, aliquoted, deep frozen in liquid nitrogen and stored at -80°C.

### Western blotting

Total protein extracts (20 μg) were denatured at 95°C for 10 min, electrophoresed in 12% gels by SDS-PAGE, and blotted onto Immun-Blot® PVDF Membrane (Biorad) as described previously [58]. The membranes were blocked with 5% nonfat dry milk diluted in TBST (20 mM Tris, 140 mM NaCl, 0.1% Tween; pH 8) for 1 h at room temperature, and subsequently incubated overnight at 4°C with the non-purified or affinity-purified Aqp0a antisera (1:1000) diluted in TBST with 1% nonfat dry milk. Horseradish peroxidase (HRP)-coupled anti-rabbit or mouse IgG secondary antibodies (1:5000; Santa Cruz Biotechnology Inc., sc-2004) were added for 1 h at room temperature, and immunoreactive bands were revealed using the Immobilon^™^ Western chemiluminescent HRP substrate (Merck Millipore). To confirm the specificity of the immunoreactive signals, duplicated membranes were probed with the aquaporin antibodies, preadsorbed with 10-fold amounts of the immunizing peptide for 1 h at 37°C, together with the alpha-tubulin antibody (1:3000) diluted as above, and subsequently incubated (1:5000) with HRP-coupled anti-rabbit and anti-mouse (Santa Cruz Biotechnology Inc., sc-2005) secondary antibodies.

### RNA extraction and real-time PCR

Total RNA was extracted from the each embryo using the RNeasy Lipid Tissue Mini Kit (Qiagen), using 2 ml of Qiazol in which 1 ng of luciferase control RNA (Promega, Ref.L4761) was added as spike. Samples were treated with DNase I, and a 150-ng aliquot reverse transcribed using QuantiTect Reverse Transcription Kit (Qiagen), following the manufacturer´s instructions, for 1.5 h at 42°C. Real-time quantitative RT-PCR (qRT-PCR) was carried out using 5 μl of SYBR Green qPCR master mix (Life Technologies Corp.), 1 μl of cDNA diluted 1:5, and 0.5 μM of each primer (specific for each *aqp0* paralog, [30]). The primer efficiencies were estimated by the generation of a standard curve for each primer pair from 10-fold serial dilutions (from 1 to 0.00001) of a pool of mixed stage 13 alevin cDNA templates, which also served to determine *aqp0* mRNA expression. Amounts of *aqp0* transcripts were corrected by the spiked luciferase amounts to avoid any bias of the RNA extraction or cDNA synthesis. Each sample was assayed in duplicate on 384-well plates using the ABI PRISM 7900HT sequence detection system (Applied Biosystems, Life technologies Corp.). The amplification protocol was an initial denaturation and activation step at 50°C for 2 min and 95°C for 10 min, followed by 40 cycles of 95°C for 15 s and 63°C for 1 min and finally a temperature-determining dissociation step was carried out at 95°C for 15 s, 60°C for 15 s, and 95°C for 15 s. All curves exhibited correlation coefficients >0.99 and an efficiency between 1.9 to 2.1. Changes in gene expression during embryogenesis were determined as amounts of *aqp0* with respect to luciferase.

### Culture of CCL 1.3 cells stably expressing aquaporins and adhesion assays

This approach was based on a protocol developed by Kumari and colleagues [17,62] with minor modifications. Non-adhesive mouse fibroblast L-cells (ATCC® CCL 1.3; CRL2648^TM^) were grown at 37°C in an atmosphere of air/CO_2_ [95:5 (v/v)] in Dulbecco´s modified Eagle´s medium (DMEM, Life Technologies) supplemented with 10% v/v fetal bovine serum (FBS, Life Technologies), 260 U/ml of penicillin and streptomycin and 2 mM L-glutamine (Life Technologies). When 70% of confluence was obtained in 6-well plates, cells were transfected using Lipofectamine® 3000 (Thermo Fisher Scientific), with 5 μg BglII-digested empty pcDNA3 vector, pcDNA3-*aqp0a1*, -*a2*, -*b1* or -*b2* or gilthead seabream (*Sparus aurata*) *aqp8bb*, as a negative control for cell adhesion, or with EX-G0297-M11 vector containing Human AQP0 (410F_C11, Tebu Bio) previously digested with XhoI, as a positive control for adhesion [17]. The following day, the medium was replaced with fresh medium containing G418 (600 μg/ml, sigma-Aldrich) for the selection of the transfected cells. After three weeks, individual clones of cells expressing pcDNA3, pcDNA3-*aqp* or M11-HAQP0 transfected cells were grown and immunofluorescence microscopy was performed as described previously [63] using non-purified antiserum against salmon Aqp0s, specific anti-Aqp8bb [58], or FLAG Antibody (M2 clone, Sigma-Aldrich) with goat Alexa-488 conjugated anti-rabbit or anti-mouse secondary antibodies (Sigma-Aldrich) to evaluate the expression of each aquaporin. The selected clones were grown and one part was trypsinized and distributed at 10^5^ cells/ml in 96 well plates (96F Nunclon Delta Black Microwell, Ref. 137101, Thermo Scientific). The following day, the DMEM medium of the coated cells was replaced by 150 μl DMEM-F12 (without phenol red) supplemented with 1% BSA, 260 U/ml of penicillin and streptomycin and 2 mM L-glutamine. The background autofluorescence (Bf) was measured using a multiwell plate reader (Infinite M200, Tecan) at excitation and emission wavelengths of 495 nm and 515 nm, respectively. Prior to the addition of the test cells, 50 μl of medium was removed from each well. The remaining cells expressing vectors pcDNA3 or pcDNA3-*aqp0*s, M11-HAQP0 or pcDNA3-*aqp8bb* were exposed to 2 μM calcein-acetoxymethyl (calcein-AM, Life technologies, C3100MP) for one hour, washed twice with PBS and incubated in DMEM-F12-1% BSA for 15 min. before trypsinization. The cells were then washed with PBS and resuspended in DMEM-F12-1% BSA to obtain 10^6^ cells/ml and 50 μl of this calcein-AM stained cell suspension was loaded onto the coated cells for one hour to allow cell adhesion. The total fluorescence (Tf) of the coated+loaded cells was measured and the cells were washed twice with PBS for 5 min under agitation at 40 rpm and with DMEM-F12 for 5 min at 40 rpm to remove non attached cells. Finally, 150 μl DMEM-F12-1% BSA was added and the fluorescence of the remaining adherent cells (Af) was measured. Results were expressed as relative adhesion (A) as calculated by A = (Af-Bf)/(Tf-Bf). All crosses (coated or loaded cells) between pcDNA3, the four pcDNA3-*aqp0*, M11-HAQP0 or pcDNA3-*SaAqp8bb* transfected cells were conducted in quadruplicate, and the entire adhesion experiment repeated twice.

### Statistics

Data are presented as the mean ± SEM and were statistically analyzed by the one- or two-way ANOVA, after log- or arcsine transformation of the data when needed, followed by the Duncan’s multiple range test. Statistical analysis were carried out using the Statgraphics Plus 4.1 software (Statistical Graphics Corp., USA). A *P* value < 0.05 was considered statistically significant.

## Results

### Developmental expression of *aqp0* increases by three orders of magnitude

**Fig 1:**
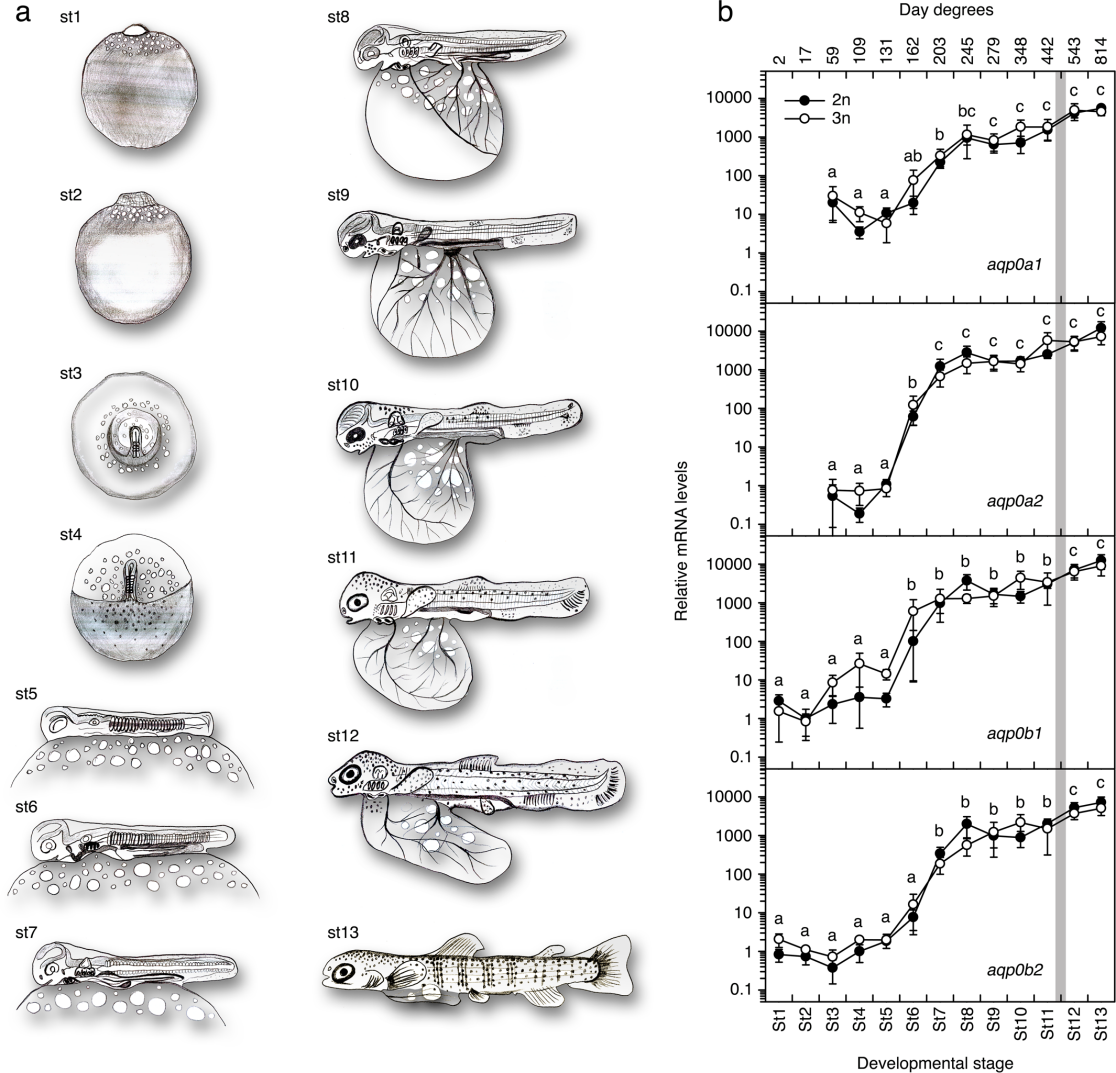
Developmental expression of *aqp0* genes in Atlantic salmon. (a) Schematic representation of the developmental stages (st) studied (based on [80], see material and methods for details). (b) Expression of *aqp0a1*, *-0a2*, *-0b1* and *-0b2* during embryonic and larval development. Gray bar behind graphs indicates the hatching time. Data represent mean expression values (± SEM., n = 5) normalized to luciferase spike mRNA expression from five diploid (2n) and five triploid (3n) embryos. *aqp0a1* and *aqp0a2* mRNA levels were undetectable at stages 1–3 in both 2n and 3n embryos. Values with a different superscript are significantly different (ANOVA, P < 0.05).

The developmental expression of *aqp0* was assessed at 13 stages of embryonic and larval development. The stages included formation of the blastodisc (stage1, 2 day degrees (dd)), the blastula (stage 2, 17 dd), the embryonic shield and four phases of somitogenesis (stage 3, 57 dd), including when the embryo exhibited 6 somites pairs (stage 4, 109 dd), ~20 somite pairs (stage 5, 131 dd), ~40 somite pairs (stage 6, 162 dd), and ~60 somite pairs (stage 7, 203 dd). Subsequent stages included extension of the vitelline plexus to 50% of the yolk surface when eye pigmentation is visible (stage 8, 245 dd), 5/6 of the yolk surface when the eyes are fully pigmented (stage 9, 279 dd), and 100% yolk-sac vascularization, then the development of 3 fin rays (stage 10, 348 dd), 10 caudal fin rays (stage 11, 443 dd), 20 caudal fin rays and the appearance of lepidotrichia in the anal and dorsal fins. Two post-embryonic stages included the newly hatched alevin (stage 12, 543 dd) and when the parr markings (pigment cells) were observed in start-feeding alevins (stage 13, 814 dd) (Fig 1A). The mRNA expression for the four *aqp0* paralogs was studied by means of qRT-PCR using specific oligonucleotide primers [30]. Transcripts for *aqp0a1* and -*0a2* were detected at low levels from stage 3 somitogenesis and drastically increased by three orders of magnitude at stage 6–7 to reach a plateau from stage 8 onwards (Fig 1B). A similar pattern was observed for *aqp0b1* and -*0b2*, although the expression of these two paralogs was detected as early as stage 1, indicating that these transcripts are maternally supplied (Fig 1B). No consistent differences were observed at the mRNA levels for the four paralogs between 2n and 3n embryos (Fig 1B), revealing that no dosage effect on *aqp0* expression was induced by the ploidy treatment.

### *aqp0a1* and *aqp0b1/2* transcripts are expressed in all three germ layer derivatives

**Fig 2:**
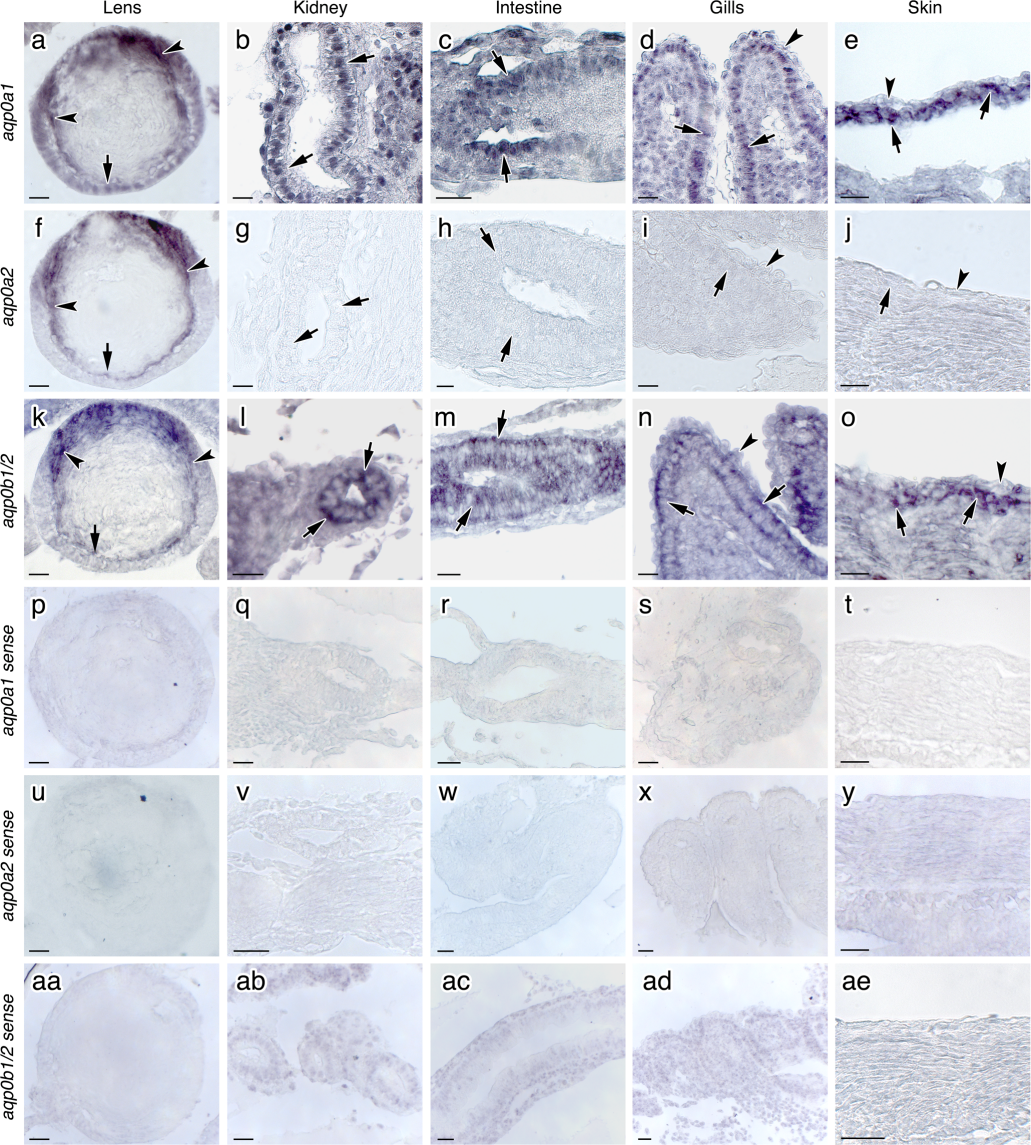
Lens and extraocular expression of *aqp0* mRNAs in Atlantic salmon embryos. Localization of *aqp0a1*, *-0a2* and *-0b1/2* transcripts in tissues of Atlantic salmon stage 9 embryos as revealed by *in situ* hybridization. Frontal sections of embryos were hybridized with antisense DIG-labeled riboprobes specific for *aqp0a1*, *-0a2* and for *-0b1/2*. The three probes gave positive signals in the cortical elongating fibers of the lens (a, f, k; arrowheads) while the epithelial precursor cells (arrows) and the differentiated fibers in the core of the lens gave a faint or no signal. In the developing kidney, epithelial cells of the pronephric ducts expressed both *aqp0a1* (b) and *aqp0b1/2* (l), but were negative for *aqp0a2* (g). *aqp0a1* and *aqp0b1/2*, but not *aqp0a2*, were also expressed by the epithelial cells of the intestine (arrows; c, h, m), the epithelial cells of the gill filament (arrows; d, i, n) but faintly in the gill pavement cells, (arrowheads; d, i, n). Both *aqp0a1* and *-0b1/2* were found in the epidermal basal layer (e, o; arrows) but not in the enveloping layer (e, o; arrowheads) of the developing skin, while *aqp0a2* was absent (j). Control sections stained with sense probes gave no signal in any tissue (P-AE). Scale bars, 20 μm.

Gene-specific probes were designed for *aqp0a1* and -*0a2* as their 3’UTRs are quite divergent (S1 Fig). However, the 3’UTR sequences of *aqp0b1* and *-0b2* were too similar to the *aqp0a*-type transcripts to achieve this goal. Consequently, the probe for *aqp0b1/2* was designed in the 5’UTR, which is sufficiently different from that of *aqp0a1/2* (S1 Fig). ISH was performed on 2n and 3n embryos at stages 9 and stage 11, however, no differences were observed between the ploidy groups, and the results are therefore presented from 2n embryos at stage 9. A strong positive signal of the three antisense probes was observed in the elongating fibers in the posterior part of the lens cortex (Fig 2A, 2F and 2K). By contrast, the differentiated nuclear lens fibers yielded either a faint or no signal, which was also the case for the epithelial precursor cells located in the anterior portion of the lens (Fig 2A, 2F and 2K). In stage 9 embryos, the developing kidney is formed by mesodermal epithelial cells delimitating the pronephric ducts (Fig 2B, 2G and 2L). These cells expressed both *aqp0a1* (Fig 2B) and *aqp0b1/2* (Fig 2L) in the mesenchymal cells surrounding the ducts, but were negative for *aqp0a2* (Fig 2g). As in the kidney, *aqp0a1* and *aqp0b1/2* but not *aqp0a2* transcripts were strongly expressed in the endodermal epithelial cells of the intestine (Fig 2C, 2H and 2M), and also in the ectodermal epithelial cells of the gill filament (Fig 2D and 2N) and the ectodermal cells forming the skin epidermal basal layer (Fig 2E and 2O), but were not expressed in the gill pavement cells (Fig 2D and 2N) or in the skin enveloping layer (Fig 2E and 2O). In turn, *aqp0a2* transcripts were absent from both gills and skin (Fig 2I and 2J). Control sections exposed to the sense probes gave no signal in any tissue (Fig 2P–2A and 2E). Similar results were obtained in 3n siblings and also in stage 11 embryos (data not shown).

### Aqp0 protein titres are not affected by ploidy status

**Fig 3:**
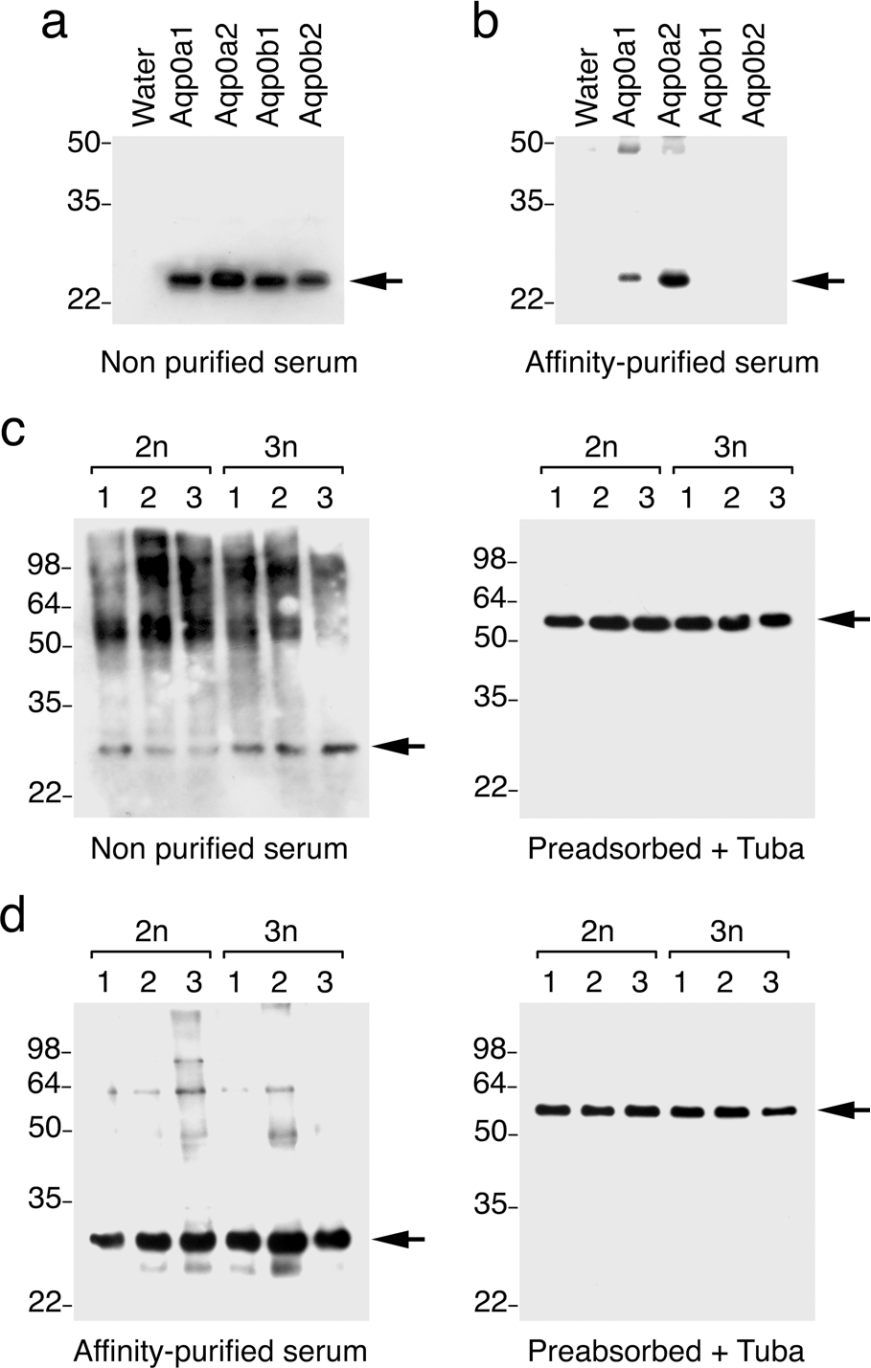
Characterization of specific anti-Aqp0 antiserum and protein expression in 2n and 3n Salmon embryos. Western blot analyses of total membrane protein extracts from *X*. *laevis* oocytes injected with water or expressing Aqp0a1, -0a2, 0b1 or 0b2 using non-purified (a) or affinity-purified serum (b). (c) Western blot analysis of protein extract from three 2n or 3n stage 9 salmon embryos using non purified serum (left panel) and preadsorded non-purified serum together with anti-tubulin antibodies (right panel). (d) Western blot analysis of protein extract from stage 9 embryos using affinity-purified serum (left panel) and preadsorded affinity-purified serum together with anti-tubulin antibodies (right panel). The monomer Aqps are indicated by arrowheads.

Our intent was to obtain specific antibodies for the Aqp0a-type and -0b-type channels. However, the primary structures of the four Atlantic salmon Aqp0 paralogs are highly conserved with 84–87% identity between Aqp0a-type and -0b-type channels and 96% identity between the R4 duplicated Aqp0a1 and -0a2, and the Aqp0b1 and -0b2 paralogs [30]. The most antigenic peptide proved to be a 20 amino acid sequence common to the carboxy terminal region of Aqp0a-type channels, while the Aqp0b-type peptide failed to produce an antibody (data not shown). The Aqp0a-type peptide is 75% identical to the same region in the Aqp0b1/2 channels, but between 0–22.5% identical to the carboxy terminal region of the 38 remaining Atlantic salmon aquaporins, including all Aqp1 paralogs (S1 Table). The unpurified Aqp0a serum antibodies crossreacted equally with the four Aqp0 paralogs as revealed by Western blot on total membranes isolated from *X*. *laevis* oocytes independently expressing the corresponding cRNAs (Fig 3A). After affinity purification of the serum on columns bearing the Aqp0a1/2-specific peptide, however, the resulting affinity-purified antibodies only reacted with Aqp0a-type channels (Fig 3B). In order to identify possible differences in the expression patterns of the Aqp0a- and -0b-type channels, the non-purified and affinity-purified antisera were therefore used to compare the expression levels of Aqp0s in 2n and 3n salmon embryos.

In total membrane extracts from stage 11 embryos, the non-purified serum against Aqp0 immunoreacted with a ~30 kDa protein band, which is near the predicted molecular mass of the Aqp0 monomers (28.8–28.9 kDa) (Fig 3C). The serum also reacted with larger polypeptides with molecular masses between ~50 to ~100 kDa, which appeared as smears on the PVDF membranes and could correspond to Aqp0 dimers and tetramers and/or reveal some post-translational modifications of the proteins. When the serum was preadsorbed with the antigenic peptide, however, all positive bands disappeared, confirming the specificity of the reaction. No differences between 2n and 3n embryos were observed in terms of protein titre (the loading control protein was alpha-tubulin) (Fig 3C). When using the affinity-purified serum (Aqp0-specific), Western blots revealed a strong immunoreaction with the ~30 kDa band and to a lesser extent with bands ranging between 50–65 kDa (Fig 3D). It thus seems that Aqp0a-type channels are detected as monomers with few post-translational modifications while Aqp0b-type channels are mostly found as dimers/tetramers with post-translational modifications. The affinity-purified antibodies did not reveal any differences in Aqp0a-type expression level between 2n and 3n embryos. (Fig 3D).

### Aqp0 is localized in the lens, pronephric tubules and presumptive enterocytes

**Fig 4:**
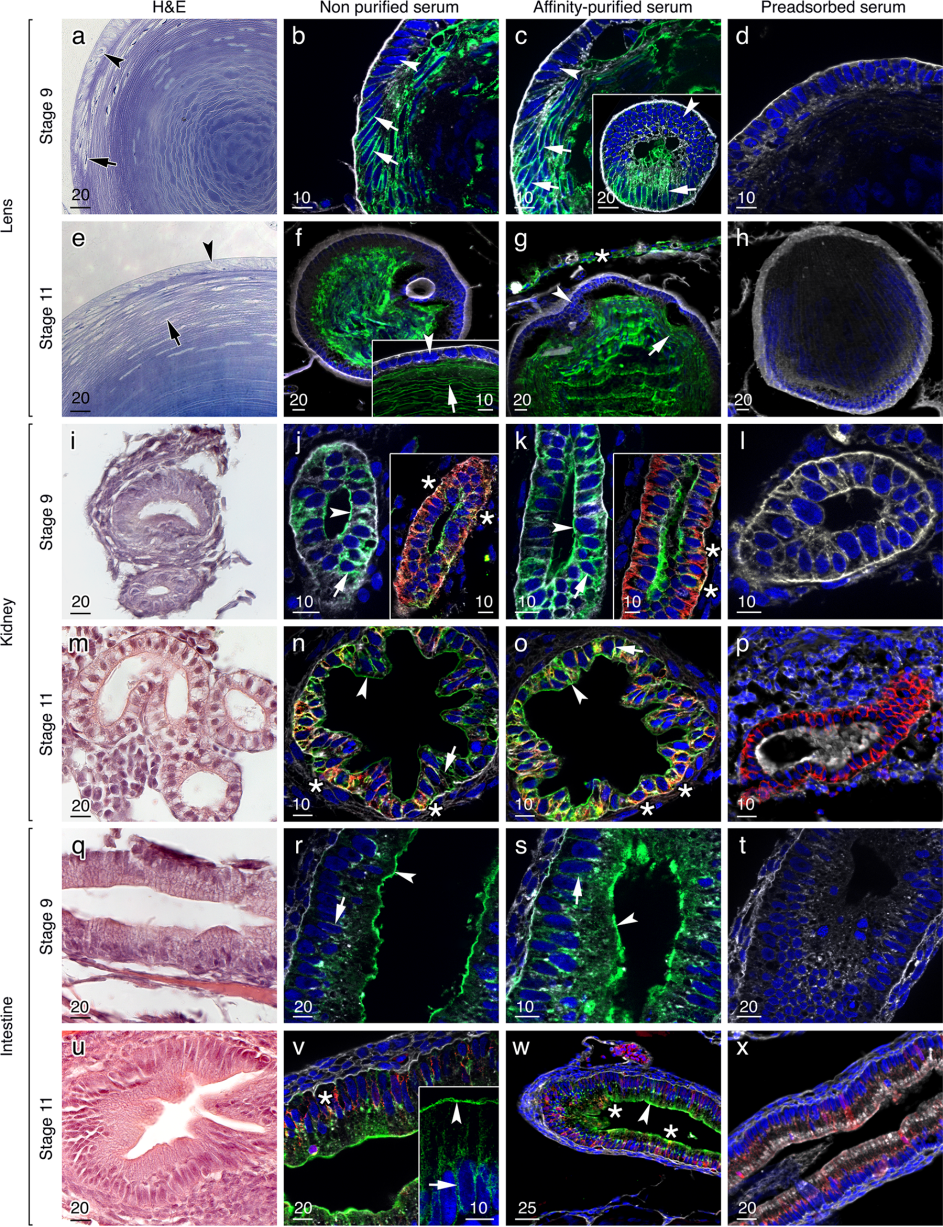
Localization of Aqp0 channels in the lens, kidney and intestine of Atlantic salmon embryos. Representative histological and immunofluorescence microscopy images of Aqp0 channel localization in stage 9 and 11 2n embryos as indicated. Histological sections were stained with either toluidine blue (a, e) or hematoxylin and eosin (i, m, q, u). Immunohistochemical sections were labeled with non-purifed and affinity-purified antibodies. The reactions were visualized with Alexa-488-coupled anti-rabbit IgG secondary antibodies (green) and the nuclei and cellular membranes (and extracellular matrix) were counterstained with DAPI (blue) and WGA (white), respectively. In some cases a Na^+^-K^+^ ATPase antibody reaction was revealed by Alexa 555-coupled anti-mouse IgG secondary antibodies (red). The same results were obtained on 3n embryos (data not shown). In stage 9 and 11 embryos, both non purified and purified sera gave a positive signal in the post equitorial elongating fibers in the lens cortex (a-c, e-g; arrows) as well as in differentiated fibers in the core of the lens but not in the epithelial precursor cells (arrowheads). In the kidney, Aqp0s were found in the apical membrane (i-k, m-o; arrowheads) and the basolateral membrane (i-k, m-o; arrows) of the pronephric epithelial cells, and colocalized in some cells with the Na^+^-K^+^ ATPase (j, k, n, o; inset, stars). A similar pattern was observed in the intestine (q-s, u-w) although Aqp0s also colocalized with some Na^+^-K^+^ ATPase expressing cells (stars). Control sections were incubated with preabsorbed non-purified antisera and no signal was observed. The same results were obtained for preadsorbed affinity-purified antibodies (data not shown). Scale bars are in μm.

The Atlantic salmon lens develops in a simlar manner to that of other teleost and mammalian embryos [64,65], where primary epithelial fibers in the anterior cortex differentiate into secondary elongating fibers toward the lens posterior that subsequently lose their nuclei and organelles as they form the inner nuclear fibers (Fig 4A and 4E). Immunofluorescence studies on stages 9 and 11 embryos using both the non-purified and affinity-purified anti-Aqp0 sera revealed strong signals in the membranes of the presumptive elongating secondary fibers found posterior to the equatorial region of the developing lens (Fig 4B and 4C and 4F and 4G). As observed for the mRNA (Fig 2), the cells of the anterior cortical lens epithelium were not labelled by either of the antisera, indicating that Aqp0 channels are not expressed in this region. By contrast, the nuclear lens fiber membranes strongly expressed the Aqp0 channels (Fig 4B and 4C and 4F and 4G). Interestingly, a strong signal was also observed in the cell membranes of the cornea (Fig 4G).

Corroborating the ISH results (Fig 2), the immunofluorescence revealed a strong expression of Aqp0 paralogs in the kidney and intestine of stage 9 and 11 embryos. In both tissues, the affinity-purified and the non-purified sera gave an intense staining in the apical membranes of some of the epithelial cells facing the lumen of the pronephric duct (Fig 4I–4P) or presumptive intestine (Fig 4Q–4X). In addition, the lateral membranes, and to a lesser extent the basal membranes of the intestinal and kidney epithelial cells accumulated Aqp0s (Fig 4I–4X), which in some cases, colocalized with the basolateral expression of Na^+^-K^+^ ATPase (Fig 4I–4X). In stage 9 embryos, however, Na^+^-K^+^ ATPase was absent in the intestine, but became evident in columnar enterocytes at stage 11, while in the kidney basolateral expression of Na^+^-K^+^ ATPase was observed in both embryonic stages. The specificities of the Aqp0 immunoreactions were demonstrated by the lack of any staining when the non-purified antiserum was preincubated with the antigenic peptide (Fig 4D, 4H, 4L, 4P, 4T and 4X). No differences were seen between the localizations of Aqp0 in 2n and 3n embryos (data not shown).

### Aqp0 channels are localized in the basolateral membranes of embryonic skin and gill epithelia

**Fig 5:**
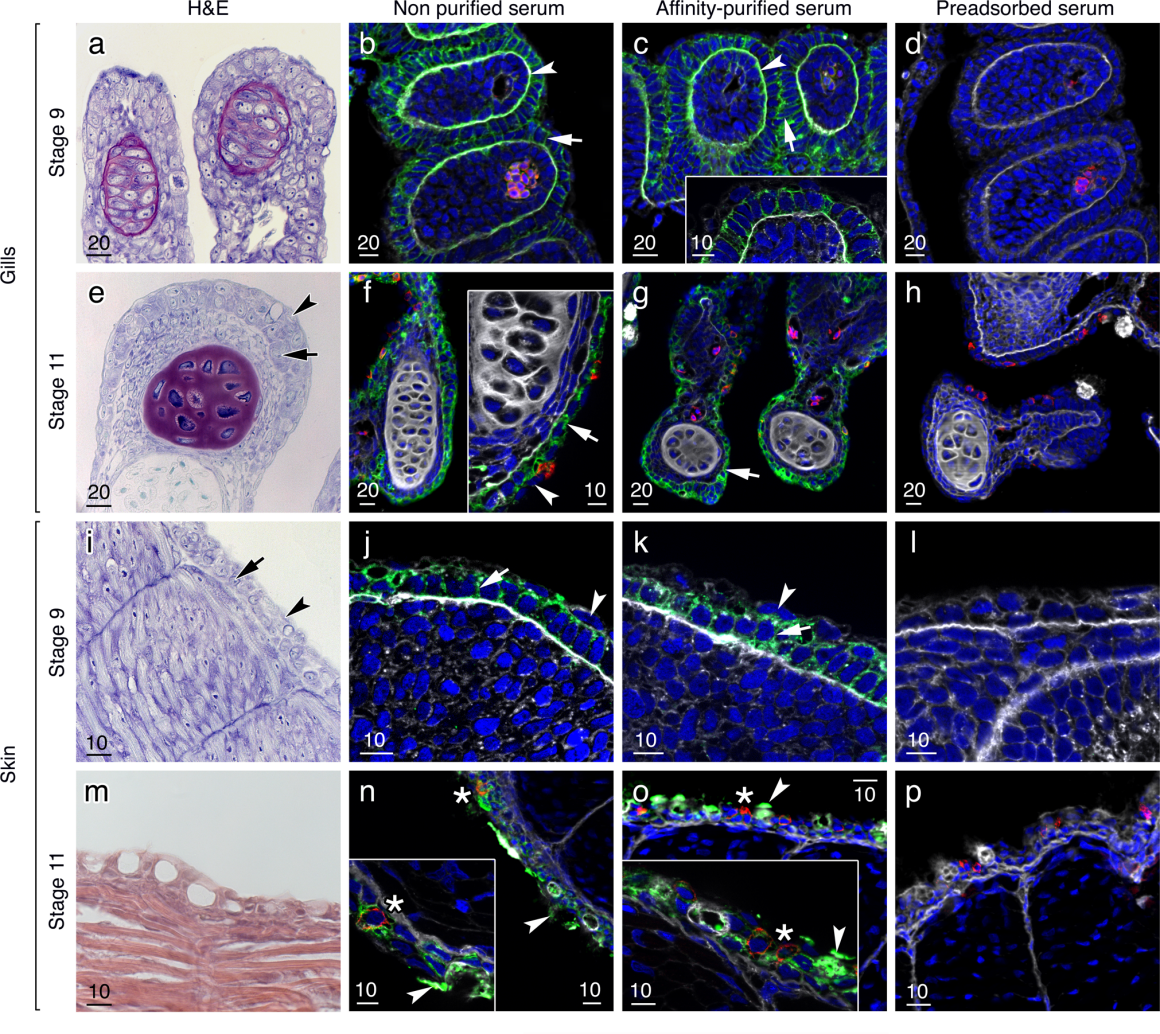
Localization of Aqp0 channels in the skin and gills of Atlantic salmon embryos. Representative histological and immunofluorescence microscopy images of Aqp0s in the gills and skin of stage 9 and 11 2n salmon embryos. Sagittal histological sections of the gills (a, e) were stained with eosin and hematoxylin revealing the cartilaginous support matrix (burgundy) of the primary filaments. Sagittal sections of the skin (i, m) were stained with toluidene blue and hemotoxylin and eosin, respectively. Immunohistochemical sections were labeled with non-purifed and affinity-purified antibodies. The reactions were visualized with Alexa-488-coupled anti-rabbit IgG secondary antibodies (green) and the nuclei and membranes (and extracellular matrix) were counterstained with DAPI (blue) and WGA (white), respectively. In some cases, a Na^+^-K^+^ ATPase antibody reaction was revealed by Alexa 555-coupled anti-mouse IgG secondary antibodies (red). In the gills of stage 9 embryos, Aqp0 channels were strongly expressed in the basal membrane (arrowheads; a-c) and the lateral membrane (arrows; a- c) of the epithelial cells of the filament. In stage 11 embryos, Aqp0s were found in basolateral and apical membranes of the gill epithelial cells (e-g; arrows), but not in the gill pavement cells (e-g; arrowheads), and did not colocalized in the Na^+^-K^+^ ATPase–positive ionocytes. In the skin of stage 9 embryos, both antisera gave a strong signal in the epidermal basal layer (i-k; arrows) but not in the enveloping layer (arrowheads), while in stage 11 embryos, Aqp0s were strongly expressed in the basolateral and apical membranes of mucus cells (m-o, arrowheads) but did not colocalize with Na^+^-K^+^ ATPase in ionocytes (stars). Control sections incubated with the preabsorbed non-purified antisera did not show any signal (d, h, l, p). The same results were obtained for preadsorbed affinity-purified antibodies (data not shown). Scale bars are in μm.

In addition to the expression in the lens, intestine and kidney, Aqp0 channels were also expressed in the epidermal epithelia of the skin and gills (Fig 5). The affinity-purified and non-purified antisera gave similar results indicating both Aqp0a-type and b-type channels are co-expressed in these tissues. In the gills of stage 9 embryos, Aqp0 was found in the basal and the lateral membranes of the filament epithelial cells (Fig 5A–5C). At this stage, there was no reaction observed with the Na^+^-K^+^ ATPase antibody indicating that branchial ionocytes are not yet differentiated. In stage 11 embryos, however, the detection of Na^+^-K^+^ ATPase in specific cells indicated that branchial ionocytes had begun to differentiate, while Aqp0 channels were distributed throughout the membranes of the gill epithelial cells, but did not seem to colocalize with the Na^+^-K^+^ ATPase positive ionocytes, and were not expressed in the pavement cells (Fig 5E–5G). In the skin of stage 9 embryos, a strong Aqp0 signal was observed in the basolateral membrane of the epidermal basal layer but not in the enveloping layer (Fig 5I–5K). No Na^+^-K^+^ ATPase positive ionocytes were observed at this stage. As in the gill, Na^+^-K^+^ ATPase was detected in selected cells in stage 11 embryos, while Aqp0 channels were strongly expressed in the basolateral and apical membranes of what appear to be putative mucus cells [66] but did not colocalize with Na^+^-K^+^ ATPase in the putative ionocytes (Fig 5M–5O). Control sections incubated with the preabsorbed non-purified antisera identified the Na^+^-K^+^ ATPase positive cells in the gills and skin of stage 11 embryos, but did not show any Aqp0 signals. The same results were obtained for preadsorbed affinity-purified antibodies (data not shown), confirming the specificity of the Aqp0 antibodies. Finally, no difference in the tissue distribitions of Aqp0 or Na^+^-K^+^ ATPase was noted between 2n and 3n embryos (data not shown).

### Salmon Aqp0 paralogs display high auto-adhesion properties

**Fig 6:**
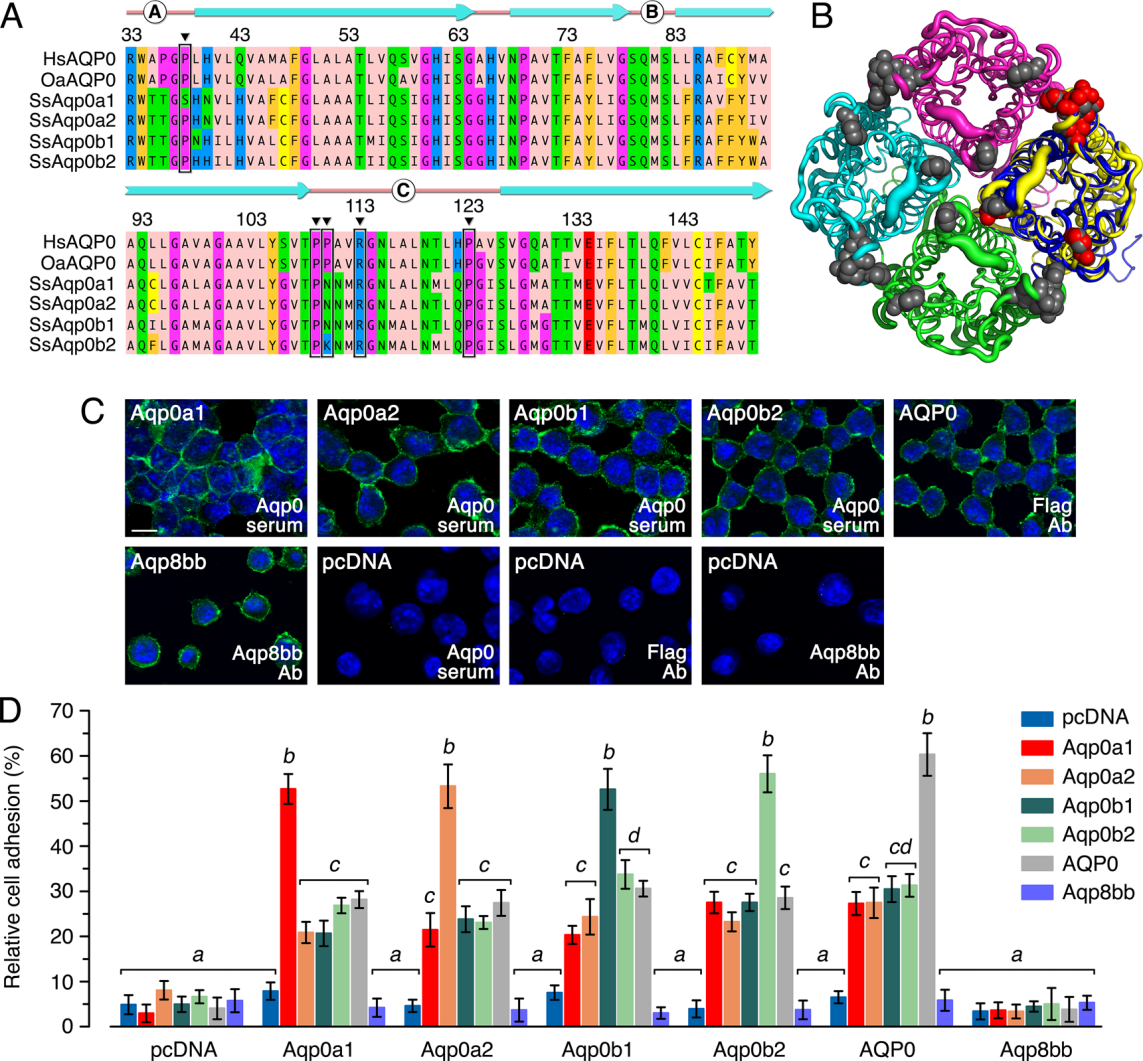
Cell-to-cell adhesion of Aqp0- expressing CCL 1.3 cells. (A) Regional amino acid alignment of a mammalian (human, HsAQP0, sheep, OaAQP0) and the Atlantic salmon SsAqp0a1, -0a2, -0b1 and -0b2 channels showing the loop (pink line) and helical (cyan arrows) domains, with interacting residues associated with cell-to-cell adhesion highlighted. (B) B-factor putty render of tetrameric OaAQP0 (green, cyan, magenta, yellow) with SsAqp0a1 (blue) wrapped to the OaAQP0 structure mask of chain 3. Interacting residues associated with cell-to-cell adhesion are shown as spacefill for OaAQP0 (grey) and SsAqp0a1 (red). (C) Immunofluorescence of CCL 1.3 cells transfected with empty pcDNA3 vector or the coding sequence of Atlantic salmon Aqp0a1, -0a2, -0b1, -0b2, human AQP0 or gilthead seabream Aqp8bb (green). Cell nuclei were counterstained with DAPI (blue). Strong immunofluorescence signals were observed at the cell-to-cell junctions. (D) Relative adhesion of calcein-AM exposed cells transfected with empty vector (control) the four salmon Aqp0s, human AQP0 (positive control) or gilthead seabream Aqp8bb (negative control) loaded onto a monolayer of cells also transfected with the empty vector or the different Aqp0 channels. The adhesion potential was measured with a microplate reader after washing. Values with a different superscript are significantly different (two-way ANOVA, *P* < 0.05).

Alignment of the four salmon Aqp0 channels to human (HsAQP0) and sheep (OaAQP0) orthologs revealed that each of the salmon channels have an ungapped amino acid identity of 68–70%. This facilitated identification of specific residues associated with octomeric adhesion [25]. Accordingly three of the residues in loop C, Pro109, Arg113 and Pro123, are fully conserved, while SsAqp0a1 encodes a Ser38 instead of a Pro38 in loop A, and the mammalian Pro110 in loop C is not conserved in the salmon orthologs (Fig 6A). A conformational wrap of SsAqp0a1 to the structure mask of OaAQP0 (3J41, [13]) confirmed the tertiary conservation of the salmon channel and the surface arrangement of the putative interacting residues (Fig 6B). To test whether the four salmon Aqp0 paralogs were able to perform cell-to-cell adhesion, we used a cell culture assay developed by Kumari and Varadaraj [17]. Adhesion-deficient mouse fibroblast L-cells were transfected with the pcDNA3 empty vector (control), gilthead seabream *aqp8bb* (negative control), human *AQP0* (positive control) or the full-length cDNA sequence of each of the four salmon *aqp0* paralogs. Stably expressing clones were obtained for each aquaporin (Fig 6C). Each of the four salmon Aqp0 paralogs and human AQP0 were strongly expressed in the plasma membrane in a pattern consistent with cell-to-cell adhesion, while gilthead seabream Aqp8bb was also expressed in the plasma membrane, but the cells were dissociated (Fig 6C). No immunofluorescent signal was observed in cells transfected with the empty pcDNA3 vector, regardless of the antisera used (Fig 6C). The adhesion assays revealed that when cells expressing pcDNA3 were used as coating or superficial layer, only 5.6 + 0.6% of Tf was observed (Fig 6D). By contrast, when cells expressing Aqp0a1, -0a2, -0b1, -0b2 or human AQP0 were used as coating or superficial layer, fluorescent cells expressing the same channel reached approximately 60% of the adhesion potential, revealing that each of the salmon Aqp0 channels have strong auto-adhesion properties (Fig 6D). Cells expressing gilthead seabream Aqp8bb did not show significantly different adhesion potential to the empty pcDNA3 controls, thus confirming the selective adhesion properties of the Aqp0 channels. Although significant interparalog adhesion was also detected for each of the salmon channels compared to the pcDNA3 controls, it was highly significantly lower than the auto-adhesive property (Fig 6D).

## Discussion

The retention of four *aqp0* genes is unusual in vertebrates, since in order to survive, it implies that each gene may have been selected for a specific function [67]. In teleosts, the duplicated *aqp0a* and *-0b* paralogs arose after the separation of the clade from the holostean fishes ~350 Ma [1,53,56,68–70], while the origin of the extra genomic duplicates in salmonids is estimated to have occurred ~80–100 Ma [1,30,71,72,73]. Since the temporal scale of duplicated gene retention is short [74], this suggests that novel functions likely arose in teleost Aqp0 channels soon after the duplication events. We have recently found that the teleost Aqp0a and -0b-type channels permeate water most efficiently at alkaline and acid pH conditions, respectively, while the permeation properties of the four salmon Aqp0 channels are uniquely sensitive to pH [30]. These observations may in part explain the retention of each gene in diploid teleosts and tetraploid salmonids. In the present context, however, we find that as in mammals, the tetraparalogous salmon Aqp0 channels are highly expressed in the developing lens, but in contrast to mammals our data reveal that at least two of the channels (Aqp0a1, -0b1 and/or -0b2) are also expressed in extraocular tissues including the epithelial membranes of the pronephros, presumptive enterocytes, gill filaments and epidermis. Quantitatively, the major increase in *aqp0* gene expression occurs between the onset of somitogenesis and pigmentation of the eyes, with no consistent differences between 2n or 3n siblings. This latter observation reveals that there is no gene dosage effect on *aqp0* mRNA expression associated with the induced triploidy, which is consistent with the absence of transcriptional differences between unstressed 2n and 3n Pacific salmon [75]. The absence of *aqp0* transcriptional differences between 2n and 3n Atlantic salmon embryos in the present study was also verifed for the proteins, which displayed similar levels in the Western blots of whole embryo extracts and identical patterns of tissue expression. Our data thus indicate that *aqp0a2* expression is lens-specific, while the other paralogs seem to have evolved additional functions beyond those associated with lens development and transparency.

The localization of the extraocular Aqp0 channels was consistent at both the mRNA and protein levels, indicating that although the antisera are not paralog-specific, they are specific for the Aqp0 channels. Thus despite reports that other Atlantic salmon aquaporins, including Aqp1aa1, -1ab2, -3a1, -8aa1/2, -8ab2, -8bb1, and 10b1/2 channels, are also expressed in the kidney, intestine and gills [76–78], the low (<23%) to non-existent identity between the antigenic peptide in the carboxy terminus of Aqp0a1/2 and these paralogs, together with the lack of cross-reactivity of the affinity-purified antibody to the Aqp0b1/2 channels, further supports the specificity of the antisera used in the present study. Consequently the striking observation that the Aqp0 channels are not only distributed throughout the basolateral membranes, but also the apical membranes of the pronephric tubules and presumptive intestinal enterocytes suggests that they may play important physiological roles in the homeosmotic control of the developing embryos. Indeed a water transport function seems plausible since the channels are not only equally efficient at permeating water at different pH levels [30], but also colocate with putative ionocytes expressing Na^+^-K^+^ ATPase. The expression of the channels at stage 9 is further consistent with the development of a hypoosmoregulatory ability of chum salmon (*Oncorhynchus keta*) embryos at the same eyed stage [79]. As in the kidney and intestinal cells, the expression of the salmon Aqp0 channels in the gills and skin was distributed in the basolateral membranes of the filament and epidermal epithelia, but did not colocalize with Na^+^-K^+^ ATPase- expressing cells. Thus besides their roles as water transporters, the teleost Aqp0 channels could also be involved in cell-to-cell adhesion.

Since the Western blots revealed major bands consistent with the full length of the Aqp0 channels, and our *ex vivo* cell adhesion experiments revealed that each channel displays strong auto-adhesive properties with respect to a given Aqp0 paralog, the present data indicate that intact salmon Aqp0 channels are capable of forming cell-to-cell junctions as found for the mammalian ortholog [17]. In contrast to mammals, however, our *in vivo* data for the embryonic lens indicate that the salmon Aqp0 channels not only perform intercellular junctions essential for fiber integrity, but may equally function as efficient water permeators. Interestingly, the lack of conservation of two of the residues, and particluarly Pro110 present in mammals, does not affect the adhesive capacity of the salmon channels, which retain an Asn or Lys in this position. Similarly, although significant interparalog adhesion can occur, the dominant property of the four salmon channels is auto-adhesion, which suggests that homo-octamers are the major constructs of the Aqp0 cell-to-cell junctions that may form in the developing lens or the pronephric, presumptive enterocyte, gill and epidermal epithelia.

In conclusion, we find that the quantitative expression of tetraparalogopus Aqp0 channels in a tetraploid teleost increases by three orders of magnitude during embryogenesis, but no gene dosage effects are associated with induced triploidy either at the mRNA or protein levels. Based upon ISH with gene-specific riboprobes and immunofluoresence microscopy with Aqp0-specific antisera and an affinity-purified antibody specific to the Aqp0a-type channels, the experiments revealed that each channel is highly expressed in the lens, while at least two of the paralogs (Aqp0a1, -0a2 and/or -0b2) are also highly expressed in epithelial tissues of the pronephros, presumptive enterocytes, gill filaments and epidermis. *Ex vivo* cell adhesion assays revealed that each channel displays strong auto-adhesive properties when expressed in adhesion-deficient mouse fibrobalst L-cells. Taken together our data suggest that intact Atlantic salmon Aqp0 channels are capable of forming cell-to-cell junctions, which represents a conserved feature in relation to the single mammalian ortholog. However, in contrast to the mammalian ortholog, the teleost channels are also efficient water transporters and may be involved in the formation of extraocular junctions in developing epithelia. While futher studies will be necessary to determine the functional implications of such junctions, the present data are the first to uncover a major extraocular role of Aqp0 in vertebrates.

## Supporting Information

S1 Fig Alignment of the 5´- and 3´-UTRs of the four Atlantic salmon *aqp0* paralogs to design primers for specific ISH probes. Alignment of the 5´UTR and 3´UTR sequences of *aqp0a1*, *-0a2*, *-0b1* and *-0b2*. Forward and reverse primers spanning the riboprobe (bold, underlined) are respectively colored in red and blue and boxed. Conserved nucleotides between *aqp0a2* and *-0b2* or between *aqp0a1* and *-0b1* or are highlighted in blue and grey in A and B, respectively.
(PDF)

S1 Table Amino acid identity of Aqp0a1/2 C-terminal immunoreactive peptide compared to other Atlantic salmon aquaporin paralogs (PDF)

## Abbreviations

Af: adherence fluorescence
AQP: aquaporin
Bf: background autofluorescence
DAPI: 4´,6-diamidino-2-phenylindole
DIG: digoxigenin
DMEM: Dulbecco’s modified Eagle’s medium
DTT: dithiothreitol
EDTA: ethylenediaminetetraacetic acid
EGTA: ethylene glycol tetraacetic acid
ELISA: enzyme-linked immunosorbant assay
FBS: fetal bovine serum
HEK: human embryonic kidney
HRP: horseradish peroxidase
IgG: immunoglobulin G
ISH: *in situ* hybridization
LLB: lens lysis buffer
PBS: phosphate-buffered saline
PBST: phosphate-buffered saline containing tween 20
PCR: polymerase chain reaction
PVDF: polyvinylidene fluoride
RT: reverse transcriptase
SEM: standard error of the mean
TBST: tris-buffered saline containing tween 20
Tf: total fluorescence
UTR: untranslated region
WGA: wheat germ agglutinin
