# Supplementary material for: Auto-Adhesion Potential of Extraocular Aqp0 during Teleost Development
Source: PLoS One. 2016 May 6;11(5):e0154592. doi: 10.1371/journal.pone.0154592 (PMC4859563; doi:10.1371/journal.pone.0154592)
Supplement: S1 Fig — Alignment of the 5´UTR and 3´UTR sequences of aqp0a1, -0a2, -0b1 and -0b2. Forward and reverse primers spanning the riboprobe (bold, underlined) are respectively colored in red and blue and boxed. Conserved nucleotides between aqp0a2 and -0b2 or between aqp0a1 and -0b1 or are highlighted in blue and grey in A and B, respectively. (PDF) [file pone.0154592.s001.pdf]

S1 Fig 5´end Alignments

```
0a2_5´        ------TAGTCCCG--TGGGCTCCCCCTGCTC---TGACAGTGAGGAT---AAATGTCTG
0a1_5´        GGCATATAGACGCACAGAGACCCACACTCCGCTCCACACAGACAGGACCAGCAGGGTTGG
0b2_5´        -----------------------------------------TCC---------GTCTATC
0b1_5´        -----AGAGAGGCTAAAACGTCCACA---------GGACAGTCC---------GTCTTTT
                                                                      *

0a2_5´        CAGGACATGCTCAACCTTTGTCTATCAATGTTCCTCTACTAACCACCAGCAGGGTTGGCA
0a1_5´        CATATAGACGCACAGAGACCCATGCTCCGCTCTACACAGACAGGACCAGCAGGGTTGGCA
0b2_5´        CCTGTCTCTA---------TTCTAGAACCACTCCTCTAGCATCAATGCACACC--TCTTG
0b1_5´        CCTGTCTCTA---------CTCTAGAACCACCCCTCTAGCATCAATGCACATC--TCCTC
              *                     *              *      *    **    *

0a2_5´        TATAGATGCACAGAGACCAACGCTCCGCTC---CACACAGACAGGA-CACAGGTCTCTAT
0a1_5´        TATAGACGCACAGAGACCCATGCTCCGCTC---TACACAGACAGGA-CGCGGTTTTCTAT
0b2_5´        ACAACTATTCCCGAGACACACACTCCCCCCACACACACACACACACCCCTATGTCTCTAC
0b1_5´        TTGACAACTATT---------------CTGAGACACACACACACACACCTATGTCGCTAC
                 *                       *      ***** ***    *     *  ***

0a2_5´        GACTAGACT-------------------AGACAGAAAGCGACGTTGCAGGTCATTGGAAG
0a1_5´        GACTGGACT----------GAAAACAGCTTACAGAAAGCGACATTGCAGATCATTGGAAG
0b2_5´        GACAGGCGGCTAGTGGAG----GCAAGCAAAGCTGAGTTGATGTTTGACTGCCTGCGCAC
0b1_5´        GACAGGCGGCCAGTGAGGGCAAGCGAGCAAATCTGAGTTGATGTTTGACTGCCTTCGC--
              ***  *                        *    *   **  **  *   * *  *

0a2_5´        TCAGTAAAGTTAGTGACTCCTAGAGAGTTGCATGTGGAACTGTTGTTTAAGTGCGGAAGC
0a1_5´        TCAGTAAAGTTAGTGACTCGTAGAGAGTTGCATGTGGAACTGTTGTTTACGTGCGGAAGC
0b2_5´        CAACTCCAGATCGTTCGTAGATTAGAAATCGATCTGT-CGGTGGATTCCTCTG--TCGAC
0b1_5´        CAACTTGAGATCGTCCGTAGCTTAGAGCTAGATCTGTATGGTGGATTCCTCTG--TCAAC
                * *  ** * **   *     ***  *  ** **         **    **      *

0a2_5´        ATCTTTAGGTTTAGAGGCACTTTTTGCTT--TTAAGTTTGCTGTCTTGTTTTTTGTTTAT
0a1_5´        ATCTTTAGGTTTAGAGGCCCATTTTGCTT--TGAAGTTTGCTCTCTTGTTTT---TTTAT
0b2_5´        TTTTTAAACTGTTTGTCTTCCAACTGATTGGCTCAGTGAGACA----ACGTTTAGGTCAG
0b1_5´        TTTTTTAACTGTTTGTCTTCCAACTGATTGGCTAAGTAAGACA----GCGTTTGGGTCAG
               * ** *  * *       *    ** **     ***  *          **    * *

0a2_5´        ACTACCTTGAAGGCA------------TCACTGAGATAGTCATTATTGATACGGTAAACA
0a1_5´        CCTACCTTGAAGGCA------------TCACTGAGATAGTCATTATTGATACGCTGAGCA
0b2_5´        CCCAGCTAGCTGACTGCCAAAGGGAAATCACTTTTTTTTTTTTTTTTTGCATCTGAAACA
0b1_5´        CCCAGCAAACTGACCTCCAAAGGGATAAAAAAAAAAAATTAAGTGCAAA-----------
               * * *     * *               *         *   *

0a2_5´        GAGCTTGGTGACTCAAAAGGAGAATACAC-ATTTTCTCCTGTTTTCGCTCCTCTCTTCAT
0a1_5´        GAGCTAGCTCACTCTAAAGGAGAATACAC-ATTTGGGGTAGCCATTTGGGCT--------
0b2_5´        GTGCAAAAAAATCACCTAGACTTTTAGAAGGTACAA----GCTTTTTCTAGATTCACCCC
0b1_5´        -------ACGAGTAACAAGACTTTTAAAACGTACACGCTTGCCTTTTCTAGATTCACCTT
                        *      **     ** *   *        *   *

0a2_5´        CCCTCCATTGCTTTG------------------------GGGTAGCATCTTGACTTTTTG
0a1_5´        -------------------------------------------AGCCTCTTGACTTTTTG
0b2_5´        AACAACTTTTTT--------CCCAAGAACATTTTCCGTTCTGAGGAGCGTCCAGTAAACG
0b1_5´        GACAACTTTTTGTTGTTGTTGAGAAAAACATTTTCTGTTAC-------AAGGAGTGAACA
                                                                  * *

0a2_5´        GCCACCATGTGGGAGTTC
0a1_5´        GCCACCATGTGGGAGTTC
0b2_5´        GCCAGCATGTGGGAGTTC
0b1_5´        GCCAGCATGTGGGAGTTC
              **** *************
```

S1 Fig 3´end Alignments

```
0b2_3´        CAAGACACAAGCCCTATAAACCTGCACCTGTCTGGGGAC---------------------
0b1_3´        CAAGACACAAGCCCTATAAACCTGCACCTGTCTGGGGAC---------------------
0a2_3´        CAAGACTCAGGCCCTATAAGCCTGGTGGAAGAAGAAACAGGACATGCTGAATTACAG---
0a1_3´        CAAGACTCAGGCCCTATAAGCCTGGT-GAAGAAGGTTGGCTTTCTGAACCCCACATTCCC
              ****** ** ********* ****

0b2_3´        ------------------------------------------------------------
0b1_3´        ------------------------------------------------------------
0a2_3´        ------------------------------------------------------------
0a1_3´        TATTCCGTACCCAAACCTCCTCCGAACCCTCAACCCTACCCAACACACATACACACACAT

0b2_3´        ------------------------------------------------------------
0b1_3´        ------------------------------------------------------------
0a2_3´        ------------------------------------------------------------
0a1_3´        TACTCTTCCTCTCTAACCCACACACCCACATAGTACACACAGACCACCACGACAACTCCA

0b2_3´        ---------------------------------AAGGGAC--------------------
0b1_3´        ---------------------------------AAGGGACGAGGGACAGGCCCTGTGTCC
0a2_3´        ----CAAGACTCAGGCCCTATAAGCCTGGTGGAAGAAGAAACAGGACATGCTGAATTACA
0a1_3´        CCTTTCTCACCCTCCTCATCCTTACTCTGCTGATCCAGAAACAGTACATGCTGAGTTACT
                                                    **

0b2_3´        ------------------------------------------------------------
0b1_3´        G------CCAACCCCACGCTGGCCCCATCCCATGAGCACATATACA---------GTCCC
0a2_3´        ------------------------------------------------------------
0a1_3´        GGGAAGCCAAAGCCCTAACCCTCCTCCGACCTGTGAA---------CTATGACCCCCTCCC

0b2_3´        ------------------------------------------------------------
0b1_3´        TCTCATTGACTCACTAACTAACACACACACCCATGTCGCTACGACAGGCGGCCAGTGAGG
0a2_3´        CCCCTTGGACTGCAGGATAAAGATGGAGGGTAAGA-AGAGGA-CTTAGTTGCCACTGAA-
0a1_3´        CCCCTTGGACTGCAGAACAAAGATGGAGGGTAAGA-AGAGGA-CTTAGTTGCCACTGAA-

0b2_3´        ------------------------------------------------------------
0b1_3´        GCAAGCGAGCAAATCTGAGTTGATGTTTGACTGCCTTCGCCAACTTGAGATCGTCCGTAG
0a2_3´        GAAAGCACCCTGCTGTGGCCTGTGGTGTAG-GGAATGGGTCACTCCTGGCTGACCAGGTG
0a1_3´        GAAAGCACCCTGCTGCGGCCTGTTGTGTAG-GGAGTGGGTGACTCCTGGCTGACCAGGTG

0b2_3´        ------------------------------------------------------------
0b1_3´        CTTAGAGCTAGATCTGTATGGTGGATTCCTCTGTCAACTTTTTTAACTGTTTGTCT----
0a2_3´        GCTATAACTGCTCAGGACAGGGGCATCTCTCTCTCTCT---------CTCTTGTTTGTAGT
0a1_3´        GCTGTAACTGCTCAGGACAGGGGCATCTCTCTCTCTTCTTTTCTCTCTATATTTTGTAGT

0b2_3´        ------------------------------------------------------------
0b1_3´        --TC--------------------------------------------------------
0a2_3´        CTGCTAGGAGTGAAGCTACAGTAGAGCAGTGGACTTCTGCATGCTTTTTAATTTTTTTCA
0a1_3´        CTGCTAGGAGTGAAGCTACAGTAGAGCAGTGAACCTCATGCTTTTTCCATTTTTTATGAA

0b2_3´        ------------------------------------------------------------
0b1_3´        ------------------------------------------------------------
0a2_3´        TCCAGTTTGAATCTCTCCCTTGTGTTTTATTGTTTTTGTTTC-ATGAATCT---------
0a1_3´        AC---TTCTGGTACCTAATGTGCATGTTATTATTATGCCCTTGAAGAATTTATGAGGATT
```

```
0b2_3´      ------------------------------------------------------------
0b1_3´      ------------------------------------------------------------
0a2_3´      -TCGGGAACCTGACATGTATGTTATATTATACCTTT----------AAAGAATTTAAAAA
0a1_3´      TTTTTCAACTCTAGTTATATTTTCAAGTCATGCATTGTGTTGTTTGTGCGTGTTTTAGAA

0b2_3´      ------------------------------------------------------------
0b1_3´      ------------------------------------------------------------
0a2_3´      AAAAAAAAAAAAAAAAAAAAAAAAAAAAAAAAAAAAAAA---------------------
0a1_3´      GACCATTGTTATTTTTATTTTATTTTTTTCATTAACAATTCCTTTGTTCATTATCATGCT

0b2_3´      ------------------------------------------------------------
0b1_3´      ------------------------------------------------------------
0a2_3´      ------------------------------------------------------------
0a1_3´      TTATTTACAATGACAGCCTACCAGGGAACAGTGGGTTAACTGCCTTGTTCAGTGACAGAA

0b2_3´      ------------------------------------------------------------
0b1_3´      ------------------------------------------------------------
0a2_3´      ------------------------------------------------------------
0a1_3´      CAACAGATTTTTACCTTGTTAGACTGCAGCCTATTCTAAGGGAAAGAAGCATGTTTTGTC

0b2_3´      ------------------------------------------------------------
0b1_3´      ------------------------------------------------------------
0a2_3´      ------------------------------------------------------------
0a1_3´      CCCCTCTTCCTTCAATGAAAGTTCTGATTCATTACTAGCAACATATTTAGAGTGCTCCCC

0b2_3´      ------------------------------------------------------------
0b1_3´      ------------------------------------------------------------
0a2_3´      ------------------------------------------------------------
0a1_3´      TTTTGTTCAAAAGCCCTTACATTTTTGCCGTCTTTTTAAAAATGGTTCTGTCTAATTGTA

0b2_3´      ------------------------------------------------------------
0b1_3´      ------------------------------------------------------------
0a2_3´      ------------------------------------------------------------
0a1_3´      GACTGTCTCTTCTAGGGGAGCACAAACAGTCAGATTTATATGAAAATAAACACCCAACAC
```
