# Supplementary material for: Auto-Adhesion Potential of Extraocular Aqp0 during Teleost Development
Source: PLoS One. 2016 May 6;11(5):e0154592. doi: 10.1371/journal.pone.0154592 (PMC4859563; doi:10.1371/journal.pone.0154592)
Supplement: S1 Table — (PDF) [file pone.0154592.s002.pdf]

S1 Table: Amino acid identity of Aqp0a1/2 C-terminal immunoreactive peptide compared to other Atlantic salmon aquaporin paralogs

Paralog	% identity
Aqp0a1/2	100.0
Aqp0b1/2	75.0
Aqp1aa1/2	22.5
Aqp1ab-1/2	20.0
Aqp15-1/2	20.0
Aqp4a1/2	5.0
Aqp4b1/2	10.0
Aqp14	0.0
Aqp8aa1/2	0.0
Aqp8ab1/2	0.0
Aqp8ba1/2	0.0
Aqp8bb1/2	0.0
Aqp3a1/2	2.5
Aqp3b1/2	2.5
Aqp7	0.0
Aqp9a1/2	10.0
Aqp9b1/2	7.5
Aqp10a1/2	15.0
Aqp10b1/2	5.0
Aqp11a1/2	5.0
Aqp11b1/2	10.0
Aqp12-1/2	5.0
